# Women Agro-Entrepreneurship Promoting Vegetables at a Family Level: A Healthcare Approach towards Non-Communicable Disease Risk Reduction

**DOI:** 10.3390/healthcare11152165

**Published:** 2023-07-30

**Authors:** A. K. M. Shahidullah, Anisul Islam, Lynn Kendall

**Affiliations:** 1 Department of Business Administration, Memorial University of Newfoundland, Grenfell, Corner Brook, NL A2H 5G4, Canada; 2 Centre for Natural Resource Studies (CNRS), Banani, Dhaka 1213, Bangladesh; anis@cnrs.org.bd; 3 Business Program, School of Arts and Social Sciences (SASS), Memorial University of Newfoundland, Corner Brook, NL A2H 5G4, Canada; lkendall@grenfell.mun.ca

**Keywords:** agro-entrepreneurship, family health, non-communicable diseases (NCDs), women entrepreneur, vegetable production

## Abstract

The role of women in promoting the production and consumption of vegetables at a family level towards mitigating the risk of non-communicable diseases (NCDs) is crucial. Women not only select and prepare food items consumed by their families but care about the health issues of family members. As research examining this critical role of women is scant, we attempted to understand how women as agro-entrepreneurs can promote vegetables to enhance healthcare situations. A field study was conducted in a northeastern district of Bangladesh from January to June 2019, adopting qualitative–participatory approaches that involved interviews, focus groups, and workshops. The study revealed that women play a vital role in taking care of the health of the family members, while their role in planning the family diet is exclusive. However, they have limited decision-making authority in the production and consumption of vegetables, and their knowledge and perception of NCDs are limited. The results imply that with enhanced capacity of vegetable production and better knowledge of nutrition and NCDs, women can improve family dietary habits by increasing the consumption of vegetables. Therefore, building the agro-entrepreneurial capacities of women in terms of knowledge, skills, access to finance, and decision-making authority at the family level would be a significant interventional approach for increased production and consumption of vegetables. We argue that public health strategies and policies addressing NCDs should incorporate this family-centric approach by promoting agro-entrepreneurship by women who would promote the production and consumption of vegetables.

## 1. Introduction

Conceptualizing health promotion in terms of family system and gender is particularly important as women function effectively as family health managers [1]. Women are central to the health of their families, directly contributing to the nutrition of family members through the production, processing, and selection of family foods [2]. Indeed, women’s endeavour for the family is not limited to providing appropriate nutrition; they also play key roles in diagnosing and treating illness and teaching and monitoring health-beneficial practices of the family members [3,4]. Thus, their roles within families have positioned them to become health managers or promoters of overall family health [5]. As such, there is a growing interest—academically, socially, or politically—in addressing and promoting health services at a family level [6,7,8].

Family health offers a collective concept in health situations. Utilizing women’s roles within their families effectively promotes and improves overall family health [9]. Regardless of the issues surrounding the gendered nature of healthcare services in a family, a woman, i.e., a wife or a mother, is still seen as predominantly responsible for looking after the health of the family members. It is widely assumed that their beliefs and practices are most impactful in the family [10]. Thus, women’s traditional roles within families have positioned them to directly impact the overall health and well-being of their families. Growing evidence suggests that empowering women in agricultural activities through capacity building led to various health, economic, social, and other benefits for women, their households, and communities [11,12,13,14].

Women are proven productive in many agricultural initiatives, such as home gardens [15,16,17,18]. In Bangladesh, women were found to be predominantly engaged in the maintenance of horticultural and homestead vegetable production, which are proven to be effective tools of nutrition and dietary intervention [19]. Even a small vegetable garden in the homestead can provide a substantial supply of essential micronutrients and contribute to dietary diversification in family food [20,21]. Schreinemachers and colleagues [22] showed that women’s home gardens in Bangladesh positively contribute to increasing the supply of a wide range of vegetables in rural markets, which eventually helps to diversify the vegetable consumption of the local people.

The gender dimension of horticulture is somewhat different in agriculture. In horticulture, women play a significant role in production and post-production activities. They are good at nursery management, intercropping, grading, processing, packaging, and other value-added activities. However, most agricultural field crops are produced by male labour. Tripathi et al. [23] suggest that women need further knowledge, skills, and confidence apart from ownership of land resources for more active and independent involvement in vegetable and field-based agricultural production. In most developing countries, women’s wealth-holding in the family is meagre; e.g., they own no or little productive land. Such ownership status and other social and cultural factors limit their decision-making roles relating to agricultural production [24].

In rural areas, traditionally, women are primarily responsible for household food preparation and caring for children [25]. Since agriculture is the prime means of earnings, women’s ability to generate income in this sector is severely constrained by their limited engagement and control of productive physical and human capital. Moreover, women are disadvantaged relative to men with respect to assets brought to marriage [26], current productive assets, including land, livestock, and agricultural machinery [27], and human capital. Women also lag behind in education; for example, in Bangladesh, about one in three women have no schooling, compared to one in four men. Ahmed et al. [28] showed that the lack of education in adult women in Bangladesh strongly correlates with being “ultra-poor”, as 80% of adult women with no education live below half a dollar a day. Such indigent conditions lead women and children of poor households to suffer from malnutrition.

Indeed, in those societies, women do not have much say in the decisions to allocate consumption expenditures or distribute time across the home and the market [29,30]. Illiteracy and non-entitlement of property made them invisible workers [31]. On the contrary, economically better-off women with control over the resources have positive effects on a number of important outcomes, including increased agricultural productivity [32,33]. Households do not always act in a unitary manner when making decisions or allocating resources [34,35]. In fact, the gender factor influences household agricultural productivity, food security, and nutrition security. This means that men and women within households do not always have the same preferences nor pool their resources. The non-pooling of agricultural resources within the household creates a gender gap in controlling agricultural inputs, which has important implications for productivity [36]. Kilic et al. [37] suggest that redistributing inputs between men and women in the household increases productivity.

With such roles and abilities, women’s participation in overall agricultural productivity is becoming crucial for enhanced household food supply, especially vegetables. In Bangladesh, knowledge and skill training for women in horticulture production to increase their capacity for large-scale productivity (beyond homestead) resulted in higher participation of women in vegetable production [38]. The involvement of women in horticulture increased vegetable and fruit production and, consequently, reduced malnutrition in the family through increased family consumption of produce [9]. Pengpid and colleagues [39] found that such increased participation led to increased supply and consumption of vegetables at the household level, which, in turn, contributes to the reduction in NCD risks.

We posit that the consumption of adequate vegetables is a key dietary habit for reducing NCD risks.

Given the critical role of women in family health and vegetable production, our research is poised to find answers to questions such as “What are women’s roles in family health and diet? What is the nature and extent of their participation in vegetable production? What barriers do women face with regard to the production and consumption activities of vegetables? How can women’s knowledge and perception of NCDs improve family health and, eventually, public health situations? With these guiding questions, the goal of this research is to investigate whether enhancing women’s agro-entrepreneurial capacities help promote vegetable production and consumption at a family level.

### Women in the Family System: A Conceptual Framework

In exploring women’s position in the family system and the significance of their roles in agricultural productivity, this study considers them as catalysts in improving family members’ health. We draw on the system’s theory [40] for this conceptual consideration, which led us to understand the family as a “whole”, comprising all the members and not by examining individuals in isolation. There has been debate in the literature about the definitions of the family and criticisms that most research focuses on the traditional nuclear family unit. However, the commonly held belief is that families are multifaceted, complex, socially located entities [41,42]. Though it is difficult to define family as a construct, it is nonetheless recognized across many cultures as the setting within which most people, at some point, live and conduct the private, personal aspects of their lives [43]. Therefore, “family system” thinking allows us to view family as a “whole”, in which all the members are interdependent and strongly influenced by the system’s structure, organization, and transactional patterns [44]. A healthy family system is organizationally complex, open, adaptive and an information processing system. It is an open system that operates in a circular pattern as the family members create a cycle of interaction that is influenced by the internal and external environment [45,46].

It is widely believed that a mother or wife in the family is the one who brings about the family dietary changes. There is a consensus that dietary change interventions are a necessary component of health promotion programs to prevent cardiovascular diseases and cancer [47,48,49,50,51,52]. Nutrition interventions that change family attitudes and habits will likely promote longer-lasting changes. Nader et al. [53] argue that dietary behaviours are especially well suited for family-based interventions as meals often involve the entire family. Other researchers confirm this resemblance in the family members’ eating behaviour, food choice, fat, and caloric intake [54,55,56]. Therefore, a family-centred strategy that refers to an approach to development in which the family is seen as the primary unit of consideration has emerged as an intervention mechanism [57].

**Figure 1:**
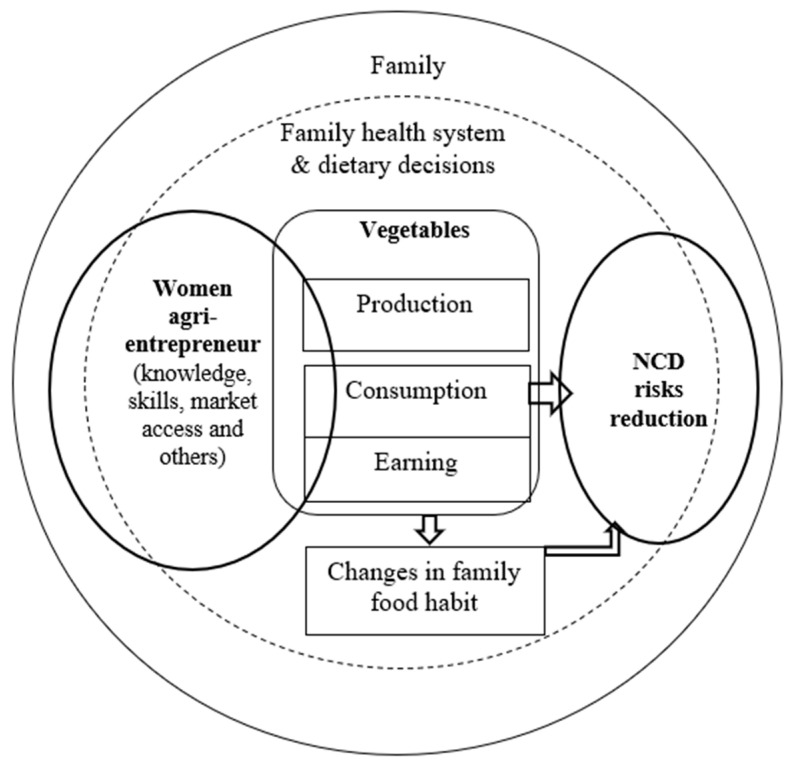
Conceptual framework exhibiting women’s agri-entrepreneurship influencing family health system and dietary decisions towards NCD risk reduction.

As delineated in Figure 1, in our study, we understand women’s agro-entrepreneurship as the function of women-led agricultural undertakings producing agricultural products both for the market and self-consumption [58]. Entrepreneurial characteristics primarily demand the possession of adequate knowledge, skills, and market access that enable a woman to undertake a vegetable venture. A portion of the produce is used for family consumption while the surplus is sold to the market for earnings that help to buy family food (including other vegetables). The women’s empowered role enables them to make family dietary decisions and change their food habits [59]. Thus, increased consumption of vegetables and enhanced family food habits will likely contribute to NCD risk reduction.

## 2. Materials and Methods

We adopted qualitative methods involving participatory approaches as data collection tools. Theoretically, qualitative participatory research is interpretive in its epistemology, acknowledging a material difference between a unit of data and its interpretation [60]. It produces plausible explanations of social functions and phenomena by encouraging a wide range of people to interpret these, including people directly experiencing the issues [61].

**Figure 2:**
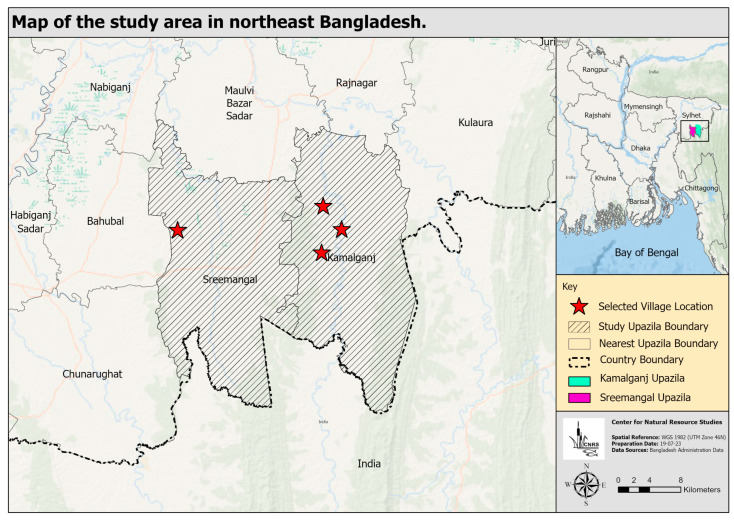
Map of the study area in northeast Bangladesh (Kamalganj, and Sreemangal upazila of Maulvibazar District).

For field exercises, we partnered with an NGO, namely, the Centre for Natural Resource Studies (CNRS). We examined one of their interventions that incorporated the promotion of women’s role in enhanced horticulture, specifically vegetable production. The intervention was made during the 2016–2018 period in two northeast districts of Bangladesh. The aim of the scheme was to increase vegetable production in the locality and, thus, eventually reduce non-communicable disease risks. We arbitrarily selected one district (Maulvibazar ) and studied four villages under the jurisdiction of two upazilas (sub-districts), namely, Kamalganj and Sreemangal (Figure 2). Our respondents primarily comprised village-based rural women agro-entrepreneurs performing key roles in the family diet and health. Fieldwork took place from January to June 2019. Data collection techniques comprised interviews with women of the household, key informant interviews (KII), focus group discussions (FGD), and workshops.

The study population comprised 150 women agri-entrepreneurs in those four villages. We selected 15 entrepreneurs (10%) for in-depth interviews based on specific selection criteria, particularly their involvement in the production of vegetables and their roles in family diet preparation and healthcare. The interview questions were semi-structured, and the interviews were conducted in-depth in order to take excerpts of the real situation in their own words (“quotes”). Other than 15 in-depth interviews, five KIIs, three FGDs, and a workshop (especially for data validation and incorporation) were held. The following table (Table 1) shows the details of the field exercises and the respondents.

The KII questions involved the locality’s vegetable production and capacity-related information. The respondents were upazila-level agriculture and horticulture extension officers, a scientific officer of Bangladesh Agricultural Research Institute (BARI), a local community organizer of the partner NGO (CNRS), and a gender specialist. Agriculture and horticulture officers and field facilitators of the project were interviewed about the support they provided to address the needs of the community farmers. The BARI scientific officer was interviewed to understand the present context with respect to resources, economic development activities, and marketing that enable women in their agro-entrepreneurship ventures.

The participants for FGDs were selected from women farmers, women HH heads, and mixed community groups (both men and women). Specific guidelines were developed to formulate the FGD questions taking local context into consideration. Both men and women within the respondent household were asked questions to ascertain their existing levels of agricultural knowledge and the nature and scale of current agricultural initiatives. Questions pertaining to gender dimensions, specifically the role of women in the production and consumption of fruits and vegetables as practiced within the community, access to land, the extent of mobility, and decision-making dynamics within households, were included in all four field exercises (interviews, KIIS, FGDs, and workshops). Community members unaware of or involved with the NGO intervention projects were also included in FGDs. This inclusion helped us gain insights from the non-beneficiary (of the project) community members, especially on how they view women’s agro-entrepreneurial roles. The NGO field facilitator helped organize the FGD sessions in sampled villages. In each FGD, there were 13 participants.

The workshop dealt with the following themes: (i) women’s role in family health and diet; (ii) women in vegetable production; (iii) women’s barriers to the production and consumption activities of vegetables; iv) women’s knowledge and perception of NCDs. It was a multi-stakeholder workshop by nature as participants (36 people) involved representatives from women farmers from study villages, NGOs, community-based organizations (CBO), local governments, and upazila-level government line agencies (horticulture, agriculture, health, and nutrition). After an introduction and general discussion of the themes by the research investigator, participants were divided into four groups (one theme for each group) to discuss and list their ideas, observations, and thoughts on the assigned theme. Later, each group presented their work to the workshop, and a consensus was reached on their findings through discussion.

Based on all four field-exercise data, we initially developed analytical notes after each interview, KII, FGD, and workshop. Data were then categorized into several key themes based on the study objective. Thus, a thematic analysis was performed that led to the development of the result chapter with a segregated theme-wise data analysis.

## 3. Results

The results of this study are grouped and interpreted thematically. We ascertained five themes in analyzing the data and presented our results based on those themes (4.1–4.5). We first presented the decision-making authority of women in their families with respect to the production of vegetables. Following this, we illustrated the role of women in the selection and preparation of the family diet. Subsequently, we tried to explain the barriers women face in their roles or activities regarding the production and consumption of vegetables. We then showed the different key healthcare-related roles that women play in their families. Finally, we tried to determine the degree of their knowledge and perception of NCDs.

### 3.1. Women in Vegetable Production

We found that women’s participation in production decisions often depends on whether they live in nuclear or extended family structures. Women make joint decisions with their husbands, but in most cases, the final decision is made by male family members. Women in the families of expatriate males, whose husbands are away for work for either short or long durations, were able to make such production-related decisions more independently than others. However, these women have limited ability and input into critical decisions such as buying lands and assets for production purposes. Most of the study participants mentioned that their involvement with the NGO-implemented project and subsequent learnings on fruit and vegetable production technologies increased the extent to which they are consulted in household decisions related to vegetable production, such as selecting seeds, saplings, and manures. A women farmer from Tilagao village of Kamalganj upazila stated:

*“We need to discuss our plans with our husbands. They give us permission to cultivate vegetables. If they do not permit us, we cannot engage in activities related to vegetable production”.*

The nature of women’s engagement in vegetable production involves activities such as the collection of quality seeds, digging soils, raising beds, ploughing, and fence making. Weeding, trimming, watering, fertilizing, and harvesting are the regular continual activities to produce most vegetables. The FGD participants and interview respondents revealed that they receive help from their husbands and, at times, grown children, especially in ploughing and digging the soil, raising beds and making fences. On most occasions, the women rely on their male counterparts for harvesting, carrying bulk quantity vegetables to market, selling produce in the local bazaar (local market), and collecting seeds from dealers.

Most of the project participants, especially women farmers, strongly agreed that NGO-led training programs helped them become knowledgeable about the science of vegetable production, which included identifying different types of vegetable seeds, determining required sizes of plots, ascertaining numbers of beds for each species, measuring plot and sowing dimensions, digging adequate beds and canals, weighing seeds, and germinating seeds using scientifically verified methods. Appreciating these learnings, Nabina from Lungpur village said:

*“We come from a farming family; we know how to cultivate vegetables. But we did not know the scientific way to prepare the bed, plant seeds, apply fertilizer, and water the plants. These new learnings will help us produce more vegetables, I believe”.*

The women farmers informed us that the project training on labour-intensive tasks, such as ploughing, digging, raising beds, and building fences, enabled them to gain production confidence. Most of them specified that the newly acquired knowledge about the line-sowing system of farming and home gardening has paid off substantially. The practice of that scientific technique has not only increased their yield but also improved the quality of the harvest. Earlier, they practiced the broadcasting system for homestead cultivation, which is perceived as a cause of inferior yields. Some of the neighbouring community members who were not project participants adopted such techniques from them, thereby transcending the benefit across the community. Most of the respondents stated that they were also engaged in dairy as part of their agricultural activities or to sustain their agricultural initiatives, including vegetable production. To rear the livestock, many women help their husbands with grazing, fodder collection, feeding, cleaning the shed, and securing the livestock properly for the night.

### 3.2. Women’s Roles in Vegetable Consumption in the Family

Farming families who cultivate vegetables frequently consume their own vegetables. Those who do not grow vegetables usually buy them from the market. Some of them also collect from their neighbours or nearby open-access areas. In the studied villages, we found that they generally cultivate okra, tomato, pumpkin, bitter gourd, gourd, different spinach, and red amaranth. Though the families eat vegetables almost weekly, their intake is inadequate. The typical dietary habit of the family is to eat rice, green chilli and sometimes spinach for breakfast. Some of them take rice with vegetables and fish. It is the adults who prefer to consume vegetables. Most families cultivate vegetables in these villages, so they are usually consumed during at least one meal. All respondents stated that they enjoy potatoes, and many noted that they also like pumpkin, gourd, carrot, cabbage, cauliflower, and beans.

The children in the families do not know about the benefits of consuming vegetables. They prefer to eat eggs, meat, and fish. Sometimes they eat hand-made *roti* (bread) with vegetables and boiled or fried potatoes. However, in one of the studied villages, the participants mentioned that most eat rice with chilli and onion or vegetables twice a day because they cannot afford better meals. Some participants gave an improved account of vegetable consumption as increased production of vegetables in the locality has been helping them to eat more of these. They also occasionally exchange surpluses with their neighbours, which helps the diversity of vegetable consumption. Hanufa from Tilagao village mentioned:

*“In our area, people are healthier than in other areas because they cultivate vegetables; they can take lots of fresh vegetables with food. Sometimes they sell the surplus to the neighbours or in the market”.*

Similarly, Amena from Langurpar village narrated:

*“Earlier, we used to buy vegetables from the bazaar; nowadays, we do not need to buy these. We also trade vegetables with our neighbours—suppose we have tomatoes while our neighbours do not have them, but they have surplus bitter gourd, then we exchange our tomatoes for their bitter gourd”.*

In all families, it is basically the women who need to set the diet menu for breakfast, lunch, and dinner based on the available food items in the household. However, they are not independent in deciding what to cook, as their male counterparts often dictate the terms of family meals. Therefore, the woman selects the items per her husband’s instructions and prepares the food based on her knowledge and ideas—mostly culturally embedded and generationally inherited. She also distributes the food among family members. Lunch and dinner are the main meals in the families. She must procure food items beforehand and start cooking preparation for most of the day. On a typical day, she mainly serves the same food for both lunch and dinner. She cooks vegetables, fish, rice, and other food for their families without having a good idea about the nutritional values of the items or the proper quantity for each person. However, she does not have a supply of fish and meat frequently, as these items are not always available, or the family cannot afford them on a regular basis. As specified, on average, the woman in the family cooks fish twice or thrice a week and meat twice a month.

### 3.3. Women’s Barriers to Production and Consumption Activities

There is a common notion in most communities of Bangladesh that income earning is usually the responsibility of men. At the same time, the remaining family members, including women and children, are economically dependent on them. The study villages were not any exception to that belief. Women have limited choices other than living in these dependent conditions. We observed that such a situation is attributed to women’s relatively lower educational levels, fewer marketable skills, and lack of social acceptance of earning by women. These circumstances limit women’s employment opportunities. Such a problem is particularly acute for women in middle-income families who face the most significant social obstacles to working outside the home. Many women spend most of their time on housework. In addition, an arbitrary division of labour is also reported by the participants in the communities. Work that receives little pay is considered unimportant and is labelled “women’s work”, though such work brings significant economic benefits to the family. Thus, there is a gender-sanctioned norm whereby men are designated farmers and women are considered helpers.

The women have limited freedom to spend money, even for family needs. We found that the husband or other male members decide on resource mobilization of the family, i.e., how the family resources or assets would be used and who should have access to them. The major limiting factor is their asset holdings. Across all four study villages, the bundle of economic entitlements of the women is poor or non-existent. Land ownership is in the names of the husbands. Such proprietary entitlement presumably made the male household member a default decision-maker in productive or consumptive family resources. Thus, men take the lead role in household purchases, and many women believe that spending money for the family is in the men’s domain of responsibility. Champa from Lagur par village stated:

*“Husbands are our masters as they earn all or most part of our family income, so we cannot do anything without their consent”.*

The limited mobility of women in rural areas also contributes to their restricted access to resources. As learnt from the FGDs and workshops, women operate based on the will of their male counterparts; they must have their husbands’ permission. We found that while both men and women are involved in vegetable cultivation, women’s contribution to the production is greater than that of the male. When it came to selling production surplus at the market, the male sold the excess produce, as outside mobility by women was highly discouraged. The cultural norm in most rural and peri-urban societies in Bangladesh virtually prohibits women from going to the market to sell the produce (except in some indigenous and tribal areas). Many of these cultural norms are embedded in religious beliefs, as one woman from Sreemangal said:

*“Muslim women are not allowed to go to the market. They have the men to do it. Sending women to market is a shame in our society. It is seen as a practice which is contrary to the religious norms.”*

### 3.4. Women in Family Healthcare

This study’s participants reported that within their families, much of the daily responsibility of keeping the family healthy and dealing with everyday ailments and illnesses falls to women. Two crucial aspects concerning family healthcare by women were revealed from the FGDs. First, women carry out the activities and precautions that promote health and prevent illness. Second, they deal with ill health, including minor ailments, chronic conditions, and general and severe sickness. To capture the overall healthcare roles of the women in the family, we identified the aspects of family health where the women played their roles and obtained the following responses (Table 2) from the FGD participants and KII respondents.

### 3.5. Knowledge and Perception of NCDs

It was found that the women in the studied communities did not have a clear idea about the causes of NCDs, such as cardiovascular diseases, cancers, respiratory diseases, and diabetes. The grave picture was revealed when they stated that most of them had never checked the glucose level in their blood or they had never had a medical test. We briefed the study participants about NCDs and explained that they are usually caused by lifestyle factors. Women realized that foods, diet, and nutritional status, including overweight and obesity, are the causes of NCD occurrences. They recognized the importance of a balanced diet or intake of proper food and proportion of foods to remain relatively safer from NCDs.

It was also found that the community members knew the name of diseases, such as diabetes and hypertension, which are common in the villages. Still, they do not exactly know the reasons for their occurrence. Some of them have wrong information about obesity. They argued that obesity is not a disease; instead, eating more food and becoming obese makes a person healthy. Most villagers mentioned that they have hypertension but do not regularly take medication or monitor their blood pressure. They visit the local physician to check their blood pressure only when they feel sick or suffer from severe neck pain and headache. For treatment of diabetes, they prefer to go to a personal physician as the government health complex is far away from their village. Some FGD participants stated that they were somewhat aware that physical labour, morning walks, and a balanced diet reduced diabetes. However, they were not aware that to control diabetes, they should consume more vegetables. The participants claimed that there were few occurrences of NCDs in their area. Many participants believed that introducing chemical fertilizers and pesticides in agriculture caused diseases, including blood pressure and diabetes.

Both in the workshop and FGDs, the participants were asked whether they received information on NCD through electronic media, such as radio or television. Specifically, they were asked about TV programs on the usefulness of eating indigenous vegetables and fruits. The NGO implemented an intervention project that telecasted a six-month-long weekly cooking program on various preparations with indigenous vegetables. The purpose of broadcasting the program was to build awareness among the community about consuming vegetables and fruits to reduce NCDs. All women participants in FGDs reported that they did not watch it either because they did not have access to a television or were preoccupied with household activities. Moreover, many of the participants informed us that they did not know about the program.

## 4. Discussion

Our findings confirm that the family diet is selected and processed by women. This indicates that any intervention emphasising dietary habits and family health needs to provide women with information, knowledge, skill, and freedom to make dietary-related decisions. The data showed that women often need to obey their husbands in most decision-making on family affairs, including the selection of food items. A body of previous studies indicated that socioeconomic development and healthcare approaches might further subordinate women in a systematic way when they are customarily shaped in a patriarchal social context [62,63,64].

As we observed in our research, the participation of women as farmers in vegetable production challenged centuries-old dictate of gender-sanctioned and delineated divisions of labour, whereby men were designated as farmers and women were relegated to the role of helper. Following women’s engagement in the vegetable production project, community members attended demonstrations of their vegetable plots and asked those women farmers for guidance. Thus, women involved in the NGO-facilitated intervention projects have started to move forward with a new identity as a “farmer” and build on their learnings. The identity of women as farmers with new knowledge about vegetable production technologies enhanced their position within the households.

Involvement of women in agricultural schemes of developmental agencies, i.e., NGO, made positive changes in gender norms related to access to knowledge about agricultural technologies. All the female farmers have greater confidence in their abilities and introduce themselves as “farmer”. Many of these tasks were traditionally seen as “not for women job”. The women farmers appreciated these opportunities and recognized a significant shift in traditional gender roles. With their own vegetable cultivation abilities, the women are now socially introducing themselves as agro-entrepreneurs. The women now think that the composite knowledge and skills they gained have bolstered their confidence. The new identity of “farmer” has increased their perceived value and respect within their communities.

The design of the studied project embedding gender promotion in agro-entrepreneurship in an otherwise male-dominated sector made a significant intervention. The project challenged the pre-existing gendered normative system of society. Many authors argue, in a patriarchal social context, that health policies and practices should entail gendered elements with emphasis on women’s gendered activities and the exclusion of men in domestic chores, family health, and birth-control programs [65,66].

This study delineates not only the dietary roles of women in the family but also the actions of women that go beyond that of food procurement and preparation. The women in the study villages are the major contributors to the overall family health. This contribution includes ensuring that dependent family members receive proper care, especially regarding food. It is evident from the study that women can contribute to improved family health by producing and ensuring the consumption of vegetables, which ultimately help reduce the risks of NCD occurrence in the family. However, in carrying out such responsibilities, most still need to maintain their subordination (male privilege) in the family. Therefore, women’s participation in agricultural or vegetable productions without building or enhancing their capacities would instead retain women’s patriarchal subordination in favour of male privilege within the family unit.

Bangladesh’s typical rural diet is not well-balanced [67]. Vegetables are consumed in meagre amounts in the studied communities. The typical diet menu primarily includes chilli, pulses, and small quantities of fish (based on availability) with rice. Milk, milk products, and meat are consumed rarely and meagerly (amount-wise), while fruit consumption is seasonal and includes mostly local varieties. Most respondents perceived that nutrition means foods such as meat, egg, milk, and fish. They were not quite aware that vegetables and small fish contain enough body nutrients to function correctly. Their education on the benefits of vegetables and small fish developed with NGO intervention programs, especially from the courtyard meetings and discussions about nutrition by NGOs. Awareness programs on radio and television are deemed to have little impact in this respect. One of the respondents (Marufa) from the village of Sreemangal upazila quoted:

*“When there was a CREL program, one apa from CREL came here and organized a courtyard meeting with us. We learned from her that all the green spinach should be washed or cleaned before cutting it, and the whole part of small fish should be cooked.”*

Marufa earlier participated in an intervention project entitled CREL and learned nutrition-related lessons from the NGO field representative (referred to as ‘apa’–meaning sister).

Apart from food or diet, dealing with family health situations seems gendered, with women seen as more likely to care for ill family members regardless of whether they work outside the home [68]. Lagerlov et al. [69] found that the mother believed it was her role to deal with the sick more than the father, and although sickness was also seen as a potential burden on the entire family, the impact on the family was managed by the mother. The multifaceted nature of women’s role in family health is difficult to encapsulate in a single study; however, observations suggest that women oversee family health. All the women, households, or farmers discussed or interviewed identified that they had sole or joint responsibilities for family health in providing food, passing health knowledge, nursing, and handling health crises.

Evidently, implementing a health or disease-preventing dietary agenda is very much a women’s task in the family. There has been a lack of empirical studies analyzing how dietary habits and disease risk reduction behaviours were promoted in relation to gendered normative systems in rural Bangladesh [57]. The knowledge and perception of NCDs among the communities are relatively poor. Most do not understand NCDs’ causes, nature, symptoms, and complexities. Effective communication, e.g., at a family level, in-person delivery, and in courtyard meetings by developmental agencies, helped raise awareness about NCDs. Therefore, interventions by NGOs or other development agencies would require adequately trained and capable staff with gender-transformative motivation, skills, and competencies to make the community people aware of NCDs. Awareness campaigns through electronic and mass media are helpful to an extent but not as effective and comprehensive in reaching rural communities as through face-to-face communication.

## 5. Conclusions

The role of women in promoting the production and consumption of vegetables at a family level towards offsetting the risk of non-communicable diseases (NCDs) is crucial. We posit that building agro-entrepreneurial capacities of women in terms of knowledge, skills, access to finance, and decision-making authority at the family level would promote vegetable production and improve family dietary habits and health status. Therefore, we assume that public health management of non-communicable diseases (NCDs) can benefit substantially by enabling women to gain more decision-making power, have greater access to family resources, and have better knowledge on nutrition to change family dietary habits. Women can be empowered as “farmers” who can carry out cultivation and home gardening activities independently.

This study implies that in the context of a primarily patriarchal gender order, health promotion and disease risk reduction require attention to gendered factors. It uncovered that traditional gender and religious norms in rural communities might constrain women-led agricultural initiatives. However, developmental agencies, e.g., NGOs, are deemed instrumental in bridging such gender inequalities in socioeconomic or health-related initiatives through appropriate intervention. Indeed, a high degree of awareness and knowledge about NCDs by rural people can reinforce such nutritional initiatives to redress such diseases to become more widespread and pervasive.

This study showed an effective pathway as to how women farmers can lead to positive nutritional outcomes through vegetable production. The unique contribution of this study is that it indicates that family-centric woman-led dietary habits can help reduce the risks of NCD occurrence. The dietary elements, i.e., vegetables, would be produced more if women were enabled as agro-entrepreneur or farmers (who will consume the produce and sell the surplus to the market). We argue that development interventions and public policies (especially public health policy) directed towards improved public health situations should incorporate this family-centric approach of recognizing women as the key change makers.

This study had limited scope as it was carried out in a rural setting, with almost all the respondents being rural poor. It does not reflect information or findings of a comprehensive range of social strata (for example, rich, urban, or peri-urban family systems are not included). Furthermore, because of cultural factors, there were tendencies among the respondents to hide or “remain silent” in some critical questions. The researchers had to overcome those with impromptu shadows and probing questions. Therefore, we think that further exploratory research encompassing women from all strata of society would be a comprehensive approach to informing scholarly and policy communities.

## Figures and Tables

**Table 1:** Types of field exercises, number of respondents or events, and place of the events or position of the respondent.

Tools	Respondents/Events (nos)	Villages; Category (Place/Position)
In-depth Interview	15	Tilagaon-4 Langurpar-3 Saraibari-4 Purba Layerkul-4
Key Informant Interview (KII)	5	Agricultural and Horticulture Experts (4); Gender Specialists (1)
Focus Group Discussions (FGDs)	3	(1) Tilagaon CBO (2) Saraibari and Lengurpar CBOs (3) Purba Layerkul CBO
Workshop	1	Kamalganj upazila

**Table 2:** Healthcare roles of women in the family.

Family Health Aspects	Women’s Roles
Providing health with food provisioning	The women prepare food for their families; They are responsible for processing and cooking and timely serving of food to the family members.
Surveillance and nursing	Women are the key to nurse their family members when they are ill. They are the ones whom the family members rely on while unwell.
Health knowledge dissemination	The women often teach other family members about health and healthy food; Most of their teaching is based on intergenerational learning; they try to pass on whatever they learned from elders.
Mediating with health professionals	Women at times mediate with the health professional about the remedial of family members, though the male family members mostly do it.
Dealing with health crises	Women not only deal with emergency health crises of the family members; they are the ones who also turn to relatives and neighbors for help.

## Data Availability

Data available to furnish.
